# Genome-based re-evaluation of the ECA75F sequence and development of a dual-probe real-time PCR assay for differentiating *Shigella* species and enteroinvasive *Escherichia coli*

**DOI:** 10.1128/spectrum.03988-25

**Published:** 2026-06-15

**Authors:** Junko Isobe, Tetsuya Harada, Emi Maenishi, Kazuki Saito, Keiko Kimata, Jun-ichi Kanatani, Kazunori Oishi, Sunao Iyoda, Masanori Watahiki

**Affiliations:** 1 Department of Bacteriology, Toyama Institute of Health 73984, Imizu, Toyama, Japan; 2 Department of Bacteriology I, National Institute of Infectious Diseases 13511, Shinjuku, Tokyo, Japan; 3 Division of Microbiology, Osaka Institute of Public Health 91397 , Osaka-City, Osaka, Japan; Indiana University School of Medicine Pathology and Laboratory Medicine, Indianapolis, Indiana, USA

**Keywords:** *Shigella *species, enteroinvasive *Escherichia coli*, ECA75F sequence, dual-probe real-time polymerase chain reaction, multilocus sequence typing

## Abstract

*Shigella* species and enteroinvasive *Escherichia coli* (EIEC) have highly similar biochemical, serological, and pathogenic profiles, making their differentiation challenging. Although the 25-base sequence ECA75F within the 16S rRNA gene has been used as a potential marker in PCR-based differentiation methods, some environmental *Shigella* spp. closely related to *E. coli* possess this sequence, while it remains absent in clinical *Shigella* spp. Hence, we aimed to further explore the relationships between *E. coli* and *Shigella* spp. employing multilocus sequence typing (MLST)-based phylogenetic analysis of 42 clinical *Shigella* spp. isolates and 32 *E. coli* isolates, among which 28 were classified as EIEC. *Shigella* isolates clustered within specific clades, whereas *E. coli* isolates, including EIEC, formed largely distinct clusters with limited overlap. Three O-untypable EIEC isolates clustered within the *Shigella* ST245 clonal complex (CC245), highlighting the complexity of classification within this group. Additionally, a dual-probe real-time PCR (DpRT-PCR) assay targeting both the ECA75F and the invasion plasmid antigen H gene (*ipaH*) was developed. This assay demonstrated sensitivities and specificities of 100% versus 96.7% for *Shigella* spp. and 88.3% and 100% for EIEC, respectively. Overall, these findings demonstrate that the DpRT-PCR assay provides a rapid and effective method for differentiating *Shigella* spp. from EIEC. Furthermore, for cases with ambiguous genetic profiles, such as EIEC isolates grouped with CC245, MLST-based phylogenetic analysis may provide additional resolution and clarity.

**IMPORTANCE:**

*Shigella* and enteroinvasive *Escherichia coli* (EIEC) cause clinically indistinguishable diarrheal diseases, yet they differ in surveillance reporting, outbreak control measures, and public health responses. However, routine laboratory identification often fails to clearly differentiate these pathogens because of their close genetic relatedness and overlapping biochemical characteristics. In this study, we performed a genome-based reassessment of the ECA75F sequence and developed a rapid and robust dual-probe real-time PCR assay that accurately distinguishes *Shigella* spp. from EIEC. This method overcomes the limitations of conventional PCR assays and provides a reliable tool for frontline laboratories. The ability to differentiate these pathogens quickly and accurately will improve case classification, guide appropriate public health interventions, and strengthen epidemiological monitoring in both endemic settings and outbreak investigations.

## INTRODUCTION

*Shigella* species are gram-negative, rod-shaped, facultative intracellular pathogens that do not ferment lactose. They cause shigellosis, a major public health concern, particularly among children in developing countries (1). In 1897, the genus *Shigella* was first defined, and it was taxonomically refined in the 1950s to include the following four species: *S. dysenteriae*, *S. flexneri*, *S. boydii*, and *S. sonnei*. Among these, *S. dysenteriae*, originally identified by Kiyoshi Shiga (2), is associated with the most severe clinical symptoms. *Shigella* spp. are characterized by a low infectious dose required for infection and are readily transmitted via person-to-person contact (3), thereby amplifying their public health impact. In recent years, *S. sonnei* has become the predominant cause of shigellosis worldwide (4). Although *S. sonnei* infections are often mild or asymptomatic, the increasing prevalence of antimicrobial resistance within this species has raised serious public health concerns (5).

In Japan, shigellosis is designated a Category III infectious disease under the National Epidemiological Surveillance of Infectious Diseases, established by the Act on the Prevention of Infectious Diseases and Medical Care for Patients with Infections in 1999 (6), mandating physicians to report all diagnosed cases. Upon notification, public health centers initiate contact tracing, and if *Shigella* spp. are isolated from patients or their close contacts, the case is officially recorded. The system enables a rapid and coordinated response, underscoring the importance of accurate pathogen identification.

A characteristic feature of the *Shigella* pathogen, which is adapted to humans and primates (7), is its large virulence plasmid, which facilitates epithelial cell invasion, and loss of this plasmid attenuates pathogenicity (8). Enteroinvasive *Escherichia coli* (EIEC), which causes a clinically similar disease, also carries a homologous plasmid crucial to its virulence. Multiple genomic studies, including DNA–DNA hybridization, multilocus sequence analysis, and whole-genome comparisons, have grouped *Shigella* spp. and EIEC under a specific pathovar within the *E. coli* lineage (9–11). Furthermore, recent advances in genome-based taxonomy have reclassified *Shigella* spp. as a subspecies of *E. coli* in the Genome Taxonomy Database (12, 13). Nonetheless, distinguishing *Shigella* spp. from EIEC remains difficult, even with high-resolution genome-based phylogenetic methods (14). Clinically, *Shigella* infections are more severe and readily transmissible than EIEC infections (15), which is one factor responsible for *Shigella* being treated as a distinct entity in public health surveillance. Traditionally, identification has relied on biochemical and serological tests, followed by PCR-based assays targeting genes such as *ipaH* (invasion plasmid antigen H) and *lacY* (16). However, the classification of some strains remains elusive. The presence of EIEC and the limited availability of commercial antisera further complicate the identification of *Shigella* spp., often rendering present diagnostic methods inadequate. Genotypic serotyping techniques, such as O-antigen gene cluster typing, have been evaluated (17) but are not yet routinely used for *Shigella* identification. In practice, certain isolates display *Shigella*-like characteristics but cannot be serotyped because of the lack of specific anti-O-antigen sera. Conversely, other isolates recovered from patients with dysentery test negative for *ipaH* by PCR and are therefore classified as *E. coli*, likely due to the loss of the virulence plasmid. Such ambiguous isolates present persistent diagnostic challenges and underscore the need for improved molecular discrimination. Although mass spectrometry has been introduced in some tertiary care hospitals (18), it lacks the resolution needed to reliably differentiate *Shigella* from *E. coli*. Currently, no PCR assay provides a rapid and reliable means of differentiating *Shigella* spp. from EIEC, highlighting the need for improved molecular diagnostics approaches.

In 2000, a key development was reported by Sabat et al. (19), who established a PCR method using the *E. coli*-specific forward primer ECA75F, targeting the 16S rRNA gene. Since then, ECA75F has been widely adopted for *E. coli* detection (20–23). Over the past two decades, advances in whole-genome sequencing (WGS) have greatly expanded the genomic repositories for both *E. coli* and *Shigella* spp. In this study, our objective was to reevaluate the ECA75F sequence annotation to assess its specificity and conservation. Based on the observation that clinical *Shigella* isolates are typically ECA75F-negative and *ipaH*-positive, we aimed to develop a real-time PCR assay to rapidly differentiate between *Shigella* and EIEC. The rationale was to develop and validate the assay using a panel of clinical isolates previously identified as *Shigella* or EIEC through biochemical, serological, and *ipaH*-targeted PCR methods and delineate phylogenetic relationships via multilocus sequence typing (MLST).

## MATERIALS AND METHODS

### 16S rRNA sequence data of prokaryotes and their usage

Prokaryotic 16S rRNA (*rrs*) sequence data were obtained from the DNA Data Bank of Japan (DDBJ) FTP site (https://ddbj.nig.ac.jp/public/ddbj_database/16S/). As of January 2024, the database contained 1,495,799 sequences, of which 976,830 exceeded 1,000 bp in length. The downloaded FASTA-formatted sequences were converted into tab-delimited format using FASTX-Toolkit version 0.0.14 (24) and subsequently imported into FileMaker Pro 16 (FileMaker, Inc.) for further analysis to identify genome-derived strains and the ECA75F sequence (Table 1).

**TABLE 1:** Re-evaluation of ECA75F sequence using 16S rRNA gene (*rrs*) extracted from genomic sequences of *Escherichia* and *Shigella* spp.

		Strain numbers with 7 copies of *rrs* genes		
Species	No. of *rrs^a^*	Total	Strains with ECA75F	
		No.	No.	%
*E. coli*	32,716	3,876	2,510^*b*^	65
*E. albertii*	560	70	62	89
*E. fergusonii*	947	69	0	0
*E. marmotae*	160	12	0	0
*Escherichia* spp*.*	1,076	9	4	44
(Total)	35,459	4,036	2,576	64
*S. boydii*	118	8	0	0
*S. dysenteriae*	116	7	3	43
*S. flexneri*	1,074	98	26	27
*S. sonnei*	458	46	1	2
*Shigella* spp*.*	195	2	0	0
(Total)	1,961	161	30	19

^*a*^ The 976,830 sequences of more than 1,000 bp length from DDBJ 16S ribosomal RNA sequence data of prokaryotes (January 2024, including 1,495,799 sequences).

^*b*^ Copy numbers of the ECA75F ranged from one to seven per strain. The most common were four (*n* = 531) and seven copies (*n* = 527), followed by three (*n* = 415), two (*n* = 342), one (*n* = 296), five (*n* = 213), and six copies (*n* = 186). Thus, the 1,366 strains of *E. coli* do not possess the ECA75F sequence at all.

### Bacterial strains

A total of 42 *Shigella* isolates, including 4 *S. dysenteriae*, 17 *S. flexneri*, 8 *S. boydii*, and 13 *S. sonnei* strains, together with 32 *E. coli* isolates (comprising 28 EIEC and 4 non-EIEC *E. coli*) were analyzed using MLST (Table 2; Table S3). The EIEC isolates comprised 6 O124, 4 O28ac, 4 O143, 3 O44, 2 O136, and one isolate each of serogroups O1, O29, O111, O125, and O164, along with 4 O-untypable (OUT) isolates. All bacterial isolates were obtained either from patients tested for shigellosis at the Toyama Institute of Health between 1981 and 2018 or from public health laboratories in the Miyazaki, Aichi, Oita, Mie, and Akita prefectures between 2004 and 2011 for bacterial species identification. All isolates were initially classified using biochemical and serological tests, as well as *ipaH*-targeted conventional PCR assays (Table 2). Notably, all 28 EIEC isolates tested positive for *ipaH* (23), with 3 isolates reacting with *Shigella* group B-specific antisera but not with *S. flexneri* type- or group-factor antisera, nor with *E. coli* O polyvalent antisera (Denka Co., Ltd., Tokyo, Japan), and were therefore classified as EIEC OUT. The 4 non-EIEC *E. coli* isolates were confirmed to be *ipaH*-negative by PCR and were excluded as EIEC but retained in this study owing to their temporal proximity to EIEC isolates and distinct epidemiological backgrounds. *Escherichia albertii* strains were used as the outgroup for phylogenetic analysis.

**TABLE 2:** List of the strains used for MLST data acquisition in this study

Species^*a*^	*Shigella*: serotype or *E. coli*: serogroup	Number of strains tested	PCR for *ipaH^b^*	Sequence type
*Shigella* (*n* = 42)				
*S. dysenteriae*	2, 3, 12	4	POS	146, 148, 273
*S. flexneri*	1b, 2a, 2b, 3a, 4a, 6, X, Y	17	POS	245, 630, 1021, 1025, 2412, 5400, t5
*S. boydii*	2, 4, 8, 16, 18	8	POS	145, 152, 243
*S. sonnei*	I, II	13	POS	145, 152, 1502
*E. coli* (*n* = 32)				
EIEC (*n* = 28)	OUT	4	POS	99, 245, 628
	O124	6	POS	6, 270
	O28ac	4	POS	270, 311
	O143	4	POS	280
	O44	3	POS	349, 394
	O136	2	POS	6, 270
	O1	1	POS	59
	O29	1	POS	270
	O125	1	POS	6
	O111	1	POS	6
	O164	1	POS	270
Non-invasive *E. coli* (*n* = 4)	OUT	2	NEG	7424, t4
	O44	2	NEG	34, 7424
Total		74		

^*a*^ Species identification was based on biochemical and serological testing, together with PCR detection of the *ipaH* gene.

^*b*^ POS, positive; NEG, negative.

To further evaluate the performance of the newly developed real-time PCR assay, additional isolates were included. These comprised 132 *Shigella* isolates (10 *S. dysenteriae*, 48 *S. flexneri*, 16 *S. boydii*, and 58 *S. sonnei*) and 28 additional EIEC isolates, resulting in a total of 174 *Shigella* and 56 EIEC isolates analyzed. In addition, 10 non-EIEC *E. coli* isolates were included, resulting in a total of 14 non-EIEC *E. coli* isolates, including 4 *ipaH*-negative strains identified among the isolates described above. To assess assay specificity, a panel of non-target enteric bacteria was also examined. This panel included 10 isolates of *E. albertii* and representative strains of other enteric genera, including *Plesiomonas shigelloides*, *Morganella morganii*, *Citrobacter* spp., *Yersinia enterocolitica*, *Salmonella enterica* serovar Enteritidis (*S*. Enteritidis), and *Enterobacter cloacae* complex. These isolates were selected to evaluate the potential cross-reactivity of the assay.

### Bacterial culture and DNA extraction

Bacterial isolates preserved in stock media, either Casiton medium (Eiken Chemical Co., Ltd., Tokyo, Japan) or MicroBank beads (Pro-Lab Diagnostics Ltd., Birkenhead, UK), were cultured by streaking onto trypticase soy agar plates (Becton, Dickinson and Company, Franklin Lakes, NJ, USA). Fresh colonies were suspended in 100 μL of 5% Chelex-100 slurry (Bio-Rad Laboratories, Inc., Hercules, CA, USA), mixed thoroughly, and heated at 100°C for 10 min to lyse the cells (25). After lysis, the mixture was centrifuged at 15,000 × *g* for 5 min, and the resulting supernatant was collected as the DNA template. DNA concentration was quantified using a NanoDrop spectrophotometer (Thermo Fisher Scientific K.K., Tokyo, Japan) and adjusted to approximately 50 ng/μL using the Tris–EDTA buffer (Nippon Gene Co., Ltd., Tokyo, Japan).

### MLST-based phylogenetic analysis of *Shigella* and *E. coli* isolates

MLST specialized for *Escherichia* and *Shigella* was performed as described previously (26). PCR amplification to generate templates for Sanger sequencing was performed in 20 µL reaction volumes containing 10 µL of 2× GoTaq Master Mix G2 (Promega K.K., Tokyo, Japan), 0.2 µM of each primer, and 2 µL of DNA template. The thermal cycling protocol consisted of an initial denaturation at 95°C for 2 min, followed by 30 cycles of denaturation at 94°C for 30 s, annealing at gene-specific temperatures for 30 s (55°C for *adk*, *fumC*, *icd*, and *purA*; 58°C for *gyrB*, *mdh*, and *recA*), and extension at 72°C for 1 min. PCR products were separated on 1% agarose gels, purified using the Montage Gel Extraction Kit (Merck Ltd., Tokyo, Japan), and used as templates for sequencing. Sequencing employed primers complementary to both strands of the PCR products. Each reaction was performed using the BigDye Terminator v3.1 Cycle Sequencing Kit (Thermo Fisher Scientific K.K.) and analyzed on an ABI 3500xL Genetic Analyzer (Thermo Fisher Scientific K.K.). The assembled sequences were submitted to the PubMLST database to determine allele profiles and MLST types. For phylogenetic analysis, sequences of the seven housekeeping genes (namely *adk*, *fumC*, *gyrB*, *icd*, *mdh*, *purA*, and *recA*) were concatenated into a single 3,423 bp sequence. Multiple sequence alignment was conducted using MAFFT version 7.505 (27), and a maximum-likelihood phylogenetic tree was generated with IQ-TREE version 2.1.4 beta (28). The resulting tree was visualized with FigTree version 1.4.4 (29). To simplify visualization and avoid redundancy, each phylogenetic label was defined by combining the sequence type (ST) with the corresponding serogroup or serotype. For example, *S. flexneri* with ST744 was labeled “ST744Sf,” and *E. coli* O44 with ST34 was denoted as “ST34E_O44.”

When MLST types did not match existing entries in the PubMLST database, provisional designations were assigned as follows: STt4 = (21, 35, 27, 6, 5, t4, 4); STt5 = (201, 4, 60, 3, 6, 6, 3); and STt6 = (10, 11, t6, 1301, 8, 8, 2). In STt4, a novel *purA* allele designated “t4” was identified and submitted to DDBJ (accession number: LC806906). Additionally, STt6 was inferred from the *S. flexneri* genome (accession number: CP058776).

**Fig 1:**
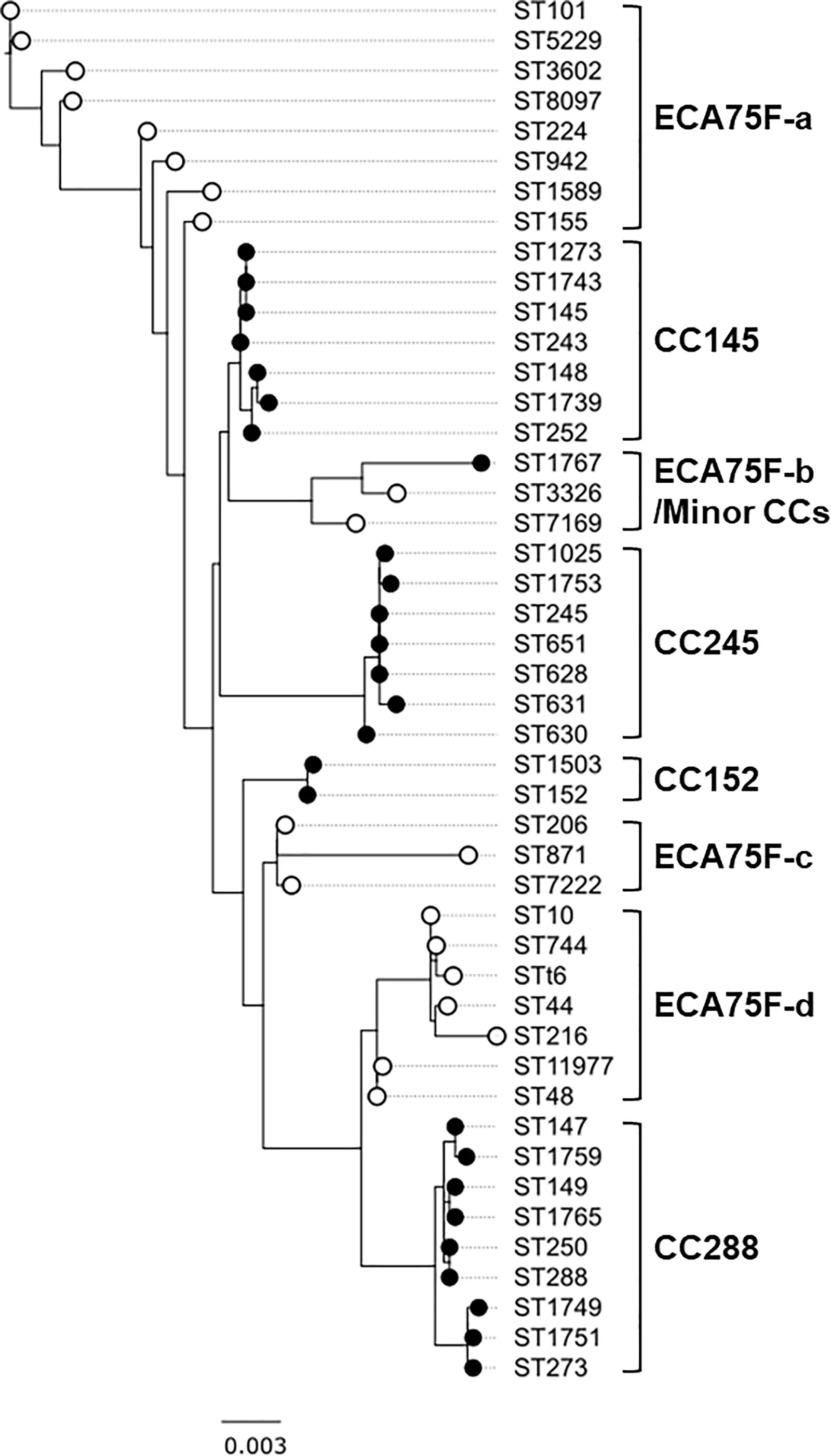
Phylogenetic tree of *Shigella* spp. based on concatenated multilocus sequence typing gene alleles (*adk*, *fumC*, *icd*, *gyrB*, *mdh*, *purA*, and *recA*), constructed using the maximum-likelihood method. White circles represent sequence types derived from genome sequences of environmental ECA75F-carrying *Shigella* strains (Table S1), whereas black circles represent STs of clinical isolates reported by Chattaway et al. (30) (Table S2). Phylogenetic clusters containing ECA75F-positive strains are indicated (ECA75F-a to -d). The ECA75F-b cluster (minorCGs) includes *Shigella boydii* ST1767 and environmental isolates annotated as *Shigella*, which are phylogenetically distinct from the major clinical *Shigella* clonal complexes. The 46 *Shigella*-derived MLST sequences used in this analysis are provided as a supplemental FASTA file containing the concatenated sequences of the seven MLST loci.

To further examine the presence of the ECA75F sequence in specific lineages identified in the phylogenetic analysis, additional BLAST analysis was performed using publicly available short-read genome data. In particular, sequence reads (100 bp) from the strain corresponding to ST1767 (30) within the ECA75F-b/minorCGs cluster (Fig. 1) were analyzed (accession number: SRR4180817; 3,095,658 reads) to assess whether the ECA75F sequence was present.

### Real-time PCR

Dual-probe real-time PCR (DpRT-PCR) was employed to simultaneously detect ECA75F and *ipaH*. Each 25 μL reaction contained 12.5 μL of Premix Ex Taq (Perfect Real Time; Takara Bio Inc., Shiga, Japan), 0.1 μM each of primers targeting ECA75F and *ipaH*, 0.2 μM of each corresponding probe, and 2 μL of DNA template.  All primers and probes were commercially synthesized (Thermo Fisher Scientific K.K.) (Table 3).

Amplification was performed on a CronoSTAR96 Real-Time PCR System (Takara Bio) under the following thermal conditions: initial denaturation at 95°C for 30 s, followed by 45 cycles of denaturation at 95°C for 5 s and amplification at 60°C for 30 s. Template DNA concentrations were normalized to ensure consistent input across assays, and reactions yielding Ct values ≤ 30 were considered positive.

DpRT-PCR results were categorized based on the presence or absence of ECA75F and *ipaH* signals into four patterns: ECA75F(+)/*ipaH*(+), ECA75F(−)/*ipaH*(+), ECA75F(+)/*ipaH*(−), and ECA75F(−)/*ipaH*(−). These signal patterns were used for the classification of bacterial isolates as described below.

### Diagnostic performance

Diagnostic performance of the DpRT-PCR assay was evaluated using conventional classification based on biochemical, serological, and *ipaH*-targeted PCR methods as the reference standard.

For performance analysis, DpRT-PCR signal patterns were interpreted as follows: isolates showing the ECA75F(−)/*ipaH*(+) pattern were considered positive for *Shigella* spp., whereas those showing the ECA75F(+)/*ipaH*(+) pattern were considered positive for EIEC. All other signal patterns were regarded as negative for the respective target group. Sensitivity and specificity were calculated using 2 × 2 contingency tables constructed separately for *Shigella* and EIEC.

## RESULTS

### *In silico* re-evaluation of the ECA75F sequence using public DNA sequence databases

Based on data obtained from a public DNA sequence database, 2,510 of 3,876 *E. coli* strains (65%) and 30 of 161 *Shigella* strains (19%) were found to contain at least one copy of the ECA75F sequence within their 16S rRNA genes (Table 1). In contrast, 1,366 *E. coli* strains (35%) lacked the ECA75F sequence. Although ECA75F was not exclusive to *E. coli* among *Escherichia* and *Shigella* strains analyzed, its prevalence was significantly higher in *E. coli* (65%) than in *Shigella* (19%) (*P* = 0.00003). This marked difference suggests that the ECA75F sequence is strongly associated with *E. coli* lineages and is largely absent from typical clinical *Shigella* strains.

The 30 ECA75F-carrying *Shigella* strains were identified solely from genome sequence data and originated from environmental rather than clinical samples (31); notably, 28 isolates were reported by a single laboratory within the same period (Table S1). Notably, all 30 strains lacked *ipaH*, which is typically present in *Shigella*. These observations suggest that the ECA75F-positive *Shigella* genomes identified in public databases represent atypical strains that differ from clinically derived *Shigella* lineages.

*In silico* MLST analysis identified 20 distinct STs, namely ST10, ST44, ST48, ST101, ST155, ST206, ST216, ST224, ST744, ST871, ST942, ST1589, ST3326, ST3602, ST5229, ST7169, ST7222, ST8097, ST11977, and the novel STt6; no sequences matched STs previously reported among clinical *Shigella* isolates belonging to major clonal complexes, such as CC145, CC245, CC152, and CC288, or the minor complexes described by Chattaway et al. (30). Phylogenetic analysis showed that these ECA75F-positive *Shigella* strains formed four distinct clusters (namely ECA75F-a, -b, -c, -d), which were clearly separated from the major clinical *Shigella* clonal complexes (CC145, CC245, CC152, and CC288) (Fig. 1). The ECA75F-b/minorCGs cluster included *S. boydii* ST1767 reported by Chattaway et al. (30) and two environmental isolates annotated as *Shigella* in public databases. To further assess the presence of the ECA75F sequence in this lineage, BLAST analysis was performed using short-read genome data of ST1767 (accession number: SRR4180817). Among 3,095,658 reads, 683 reads containing the ECA75F sequence were identified. Of these, 50 reads were randomly selected and subjected to BLAST analysis, all of which mapped to the 16S rRNA gene. Based on these findings, this strain was concluded to harbor the ECA75F sequence. This correspondence further supports the notion that ECA75F-positive *Shigella* genomes represent phylogenetically distinct and atypical lineages within the *Shigella*/EIEC complex.

Screening for *lacY* and *cadA* (32) indicated that 29 of the 30 strains carried *lacY* and all 30 carried *cadA* (Table S1). To further explore the relationship between ECA75F absence and *Shigella*-like characteristics, a BLAST search using the *ipaH* probe sequence was performed on the 1,366 ECA75F-negative *E. coli* strains (Table 1), identifying two strains (namely CP050862 and CP050865) (33). Genome analysis confirmed that these two *E. coli* strains lacked ECA75F but carried *ipaH*, and MLST assigned both to ST270. These findings suggest that, although strongly associated with *E. coli*, the presence or absence of ECA75F alone does not fully resolve the boundary between *E. coli* and *Shigella*/EIEC lineages.

### Phylogenetic analysis with MLST data of 42 *Shigella* spp. and 32 *E. coli* isolates

As the 30 ECA75F-positive *Shigella* strains (Table 1) displayed MLST sequence types distinct from those of clinical *Shigella* spp. (Fig. 1), an MLST-based phylogenetic analysis was performed (Table 2). This analysis included 42 additional *Shigella* isolates, 28 EIEC isolates, and 4 *E. coli* isolates previously obtained from patients with dysentery and previously characterized via biochemical testing, serological typing, and *ipaH* PCR assays. Among these isolates, 8 could not be definitively classified using standard procedures. Among them, 4 were designated “EIEC OUT” because they failed to agglutinate with commercially available *Shigella* spp. antisera, whereas the remaining 4 were recovered from patients with dysentery but tested negative for *ipaH* by PCR. Clarifying the phylogenetic placement of these atypical *Shigella* isolates contributes to understanding their classification and possible origins.

**Fig 2:**
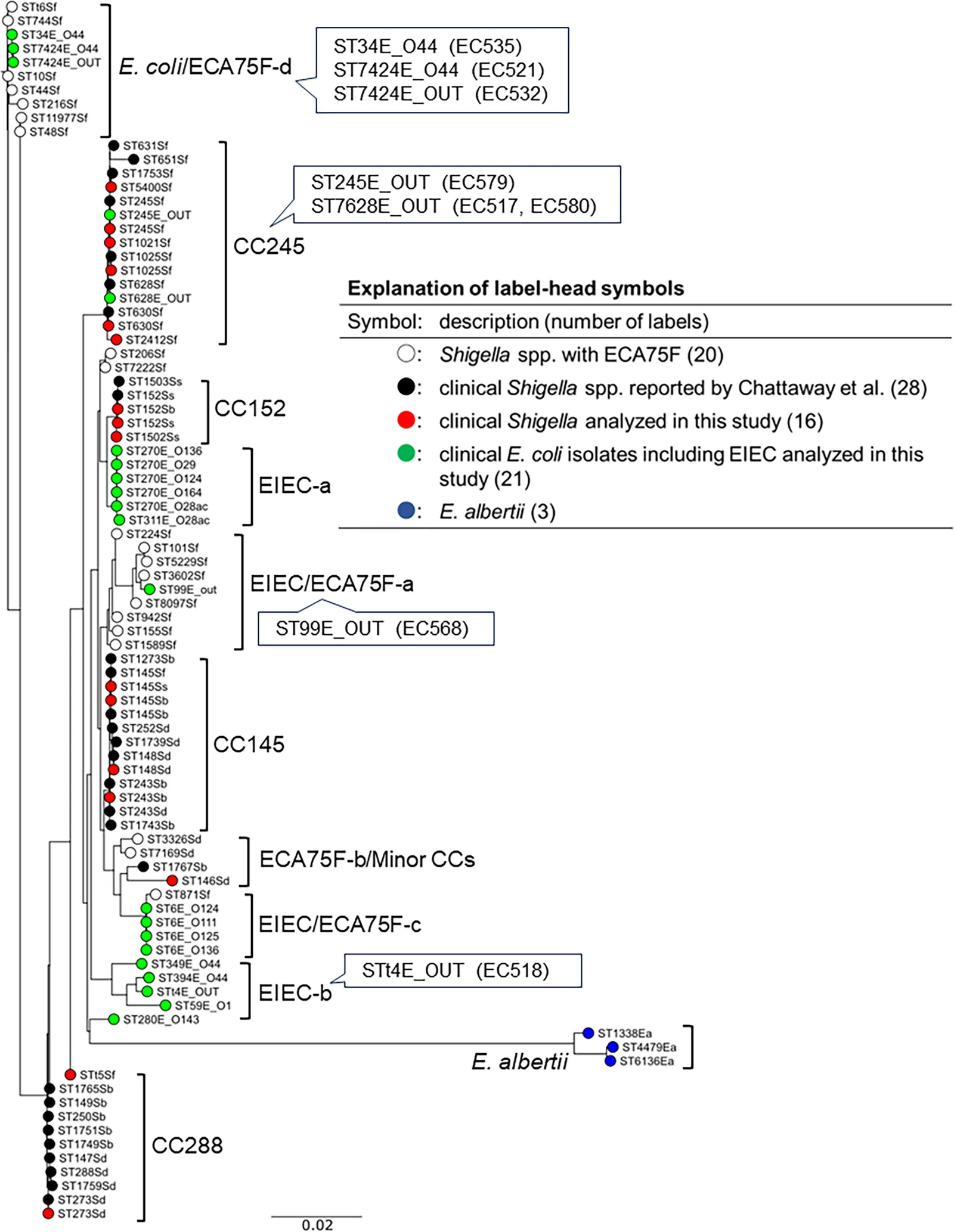
Phylogenetic tree of *Shigella* spp., EIEC, and representative *E. coli* isolates based on concatenated MLST gene sequences (*adk*, *fumC*, *icd*, *gyrB*, *mdh*, *purA*, and *recA*), constructed using the maximum-likelihood method. Strain labels indicate sequence type and serogroup/serotype. For example, “ST744Sf” denotes *Shigella flexneri* with ST744, whereas “ST34E_O44” denotes *E. coli* O44 with ST34. In several clusters, representative strain IDs for *E. coli* or EIEC isolates are shown to clarify their phylogenetic positions.

The phylogenetic tree was constructed from MLST data representing 88 labels (Fig. 2) as follows: (i) 20 ECA75F-carrying *Shigella* spp. labels; (ii) 28 clinical *Shigella* spp. labels reported by Chattaway et al. (30); (iii) 16 *Shigella* spp. and 21 *E. coli* labels (including EIEC) analyzed in this study; and (iv) 3 *E. albertii* labels, namely ST1338, ST4479, and ST6136, which served as the outgroup.

The overall tree topology revealed multiple distinct clusters, including established *Shigella* and *E. coli* clusters analyzed in this study (e.g., EIEC-a and EIEC-b), representing genetically related but largely distinct lineages with limited overlap. Although the CC245 cluster consisted predominantly of *S. flexneri*, other clusters showed no consistent association with specific *E. coli* serogroups or *Shigella* serotypes. The 16 *Shigella* labels derived from clinical isolates analyzed in this study were either assigned to one of the four established clinical clusters—CC145, CC152, CC245, and CC288, or to minor clonal complexes (Fig. 2). Conversely, the 21 *E. coli* labels, including 17 EIEC labels, were distributed across the following six clusters: ECA75F-d, CC245, ECA75F-c, EIEC-a, ECA75F-a, and EIEC-b. Among the four EIEC OUT isolates, three (namely EC517, EC579, and EC580) clustered within CC245, whereas one (EC568) was assigned to ECA75F-a. Of the four *E. coli* isolates, three (namely EC521, EC532, and EC535) grouped within ECA75F-d, whereas one (EC518) clustered with EIEC-b.

### Design of real-time PCR primers and TaqMan MGB probes

A TaqMan MGB probe, Ecoli16S-AP4-fam1, was designed based on the ECA75F sequence (5′-GGAAGAAGCTTGCTTCTTTGCTGAC-3′), originally reported by Sabat et al. (19) to be specific to *E. coli* within the 16S rRNA gene (Table 3). For specific real-time PCR amplification, primer pairs (Ecoli16S-13F and Ecoli16S-101R) were designed to target conserved regions flanking the ECA75F site. Primer and probe design was guided by complete genome sequences from nine *E. coli* and three *Shigella* strains, each containing seven copies of the 16S rRNA gene.

**TABLE 3:** Primers and probes of 16S rRNA gene (*rrs*) and *ipaH* for TaqMan probe method

Gene	Primers or probes	Sequence structure (5′−3′)	Tm^*a*^ (°C)	Amplicon size (bp)	Reference
16S rRNA	Ecoli16S-13F	CACATGCAAGTCGAACGGTA	65	88	(19) and this study
	Ecoli16S-101R	ATCAGGCAGTTTCCCAGACA	65		
	Ecoli16S-AP4-fam1	FAM-ACAGGAAGAAGCTTGCTTC-NFG-MGB^*b*^	71		
*ipaH*	ipaHf3	TAATGATACCGGCGCTCTGCTCTCC	73	174	This study
	ipaHr177	TTCCTTCACGGCAGTRGAGAGCTG^*b*^	72		
	ipaH_MProbe2r-vic	VIC-CTGCGAGCATGGTCTGGAAG-NFG-MGB^*b*^	76		

^*a*^ Tm value was calculated using Primer Express version 3.0.1 (Thermo Fisher Scientific K.K.).

^*b*^ TaqMan MGB probes, FAM and VIC, reporter dyes; NFG, non-fluorescent quencher; and MGB, Minor Groove Binder (Tm enhancer).

To design *ipaH*-targeted primers and probes, nucleotide sequences were obtained from 98 complete *Shigella* genomes and 9 plasmids available in DDBJ. Phylogenetic analysis (Fig. S1) identified a major (1,400–2,982 bp) and a minor (480–505 bp) *ipaH* group. The major group comprised chromosomal and plasmid-associated clusters, whereas the minor group formed distinct, separate clusters. Consequently, a TaqMan MGB probe (ipaH_MProbe2r-vic) and primers (ipaHf3 and ipaHr177) were designed for real-time PCR detection of all major-group *ipaH* variants. For simultaneous detection of *E. coli* and *Shigella* spp., the ECA75F probe was labeled with FAM dye (6-carboxyfluorescein), whereas the *ipaH* probe was labeled with VIC (proprietary fluorescent dye from Applied Biosystems, Inc., Carlsbad, CA, USA) (Table 3). As minor-group *ipaH* were approximately one-third the length of major-group genes, an additional TaqMan probe and primer set were designed, as described above, to investigate their potential biological significance.

### Evaluation of the performance of the DpRT-PCR assay

The 74 isolates used in the MLST-based phylogenetic analysis, comprising 42 *Shigella* and 32 *E. coli* isolates (28 EIEC and 4 non-invasive *E. coli*), were evaluated using the developed DpRT-PCR assay targeting the major group *ipaH* sequence (Table S3). In addition, 132 *Shigella* isolates and 28 EIEC isolates, and 10 non-EIEC *E. coli* isolates were tested separately. Furthermore, a panel of non-target enteric bacteria, including *E. albertii* and other enteric species, was included to assess assay specificity. In total, 174 *Shigella*, 60 EIEC, 10 non-EIEC *E. coli*, and additional non-target isolates were analyzed (Table 4).

**TABLE 4:** Specificity of the dual-probe real-time PCR assay evaluated using an expanded panel of bacterial isolates

Species/group	No. of isolates	ECA75F(+)/*ipaH*(+)	ECA75F(−)/*ipaH*(+)	ECA75F(+)/*ipaH*(−)	ECA75F(−)/*ipaH*(−)
*S. dysenteriae*	14	0	14	0	0
*S. flexneri*	65	0	65	0	0
*S. boydii*	24	0	24	0	0
*S. sonnei*	71	0	71	0	0
(S*higella* spp*.* total)	174	0	174	0	0
EIEC	60	53^*a*^	3^*b*^	4^*c*^	0
non-EIEC *E. coli*	10	0	0	10	0
*E. albertii*	10	0	0	10	0
*P. shigelloides*	1	0	0	0	1
*M. morganii*	2	0	0	0	2
*Citrobacter* spp*.*	3	0	0	0	3
*Y. enterocolitica*	3	0	0	0	3
*S*. Enteritidis	2	0	0	0	2
*E. cloacae* complex	1	0	0	0	1

^*a*^ Includes EIEC OUT isolate EC568.

^*b*^ Consists of EIEC OUT isolates EC517, EC579, and EC580.

^*c*^ Consists of *E. coli* isolates EC518, EC521, EC532, and EC535, recovered from patients with shigellosis.

Table 4 includes a summary of the specificity data for the DpRT-PCR assay analyzed using an expanded panel of bacterial isolates. All *Shigella* isolates were consistently negative for ECA75F and positive for *ipaH*, whereas EIEC isolates showed heterogeneous detection patterns, with the majority being positive for both targets. In contrast, all non-target organisms, including non-EIEC *E. coli*, *E. albertii*, *Plesiomonas shigelloides*, *Morganella morganii*, *Citrobacter* spp., *Yersinia enterocolitica*, *Salmonella enterica* serovar Enteritidis, and *Enterobacter cloacae* complex, were negative for *ipaH*, and no cross-reactivity was observed.

To evaluate diagnostic performance, 2 × 2 contingency tables were constructed separately for *Shigella* and EIEC using the conventional classification based on biochemical, serological, and PCR-based methods as the reference standard. For *Shigella,* the DpRT-PCR pattern ECA75F(−)/*ipaH*(+) was defined as test positive, whereas all other patterns were considered test negative. Under this definition, the assay showed a sensitivity of 100% (174/174) and a specificity of 96.7% (89/92). For EIEC, the pattern ECA75F(+)/*ipaH*(+) was defined as test positive. The assay demonstrated a sensitivity of 88.3% (53/60) and a specificity of 100% (206/206). These results indicate that the dual-target strategy provides high specificity for both *Shigella* and EIEC, whereas the minor heterogeneity observed among EIEC isolates likely reflects biological variation rather than cross-reactivity.

In contrast, a separate DpRT-PCR assay targeting the minor-group *ipaH* sequence yielded both positive and negative results among *Shigella* and EIEC isolates, but no consistent pattern or correlation was observed with either group, indicating that this target was not suitable for diagnostic discrimination.

## DISCUSSION

Previous studies employing DNA–DNA hybridization, MLST, and whole-genome analyses have demonstrated that *Shigella* spp. and EIEC constitute a distinct pathovar within *E. coli* (9–11). However, their close genetic relatedness continues to complicate clinical differentiation. To address these limitations, we initially developed an endpoint PCR assay targeting both *ipaH* and ECA75F, a sequence located in the V1 region of the 16S rRNA gene that was previously described by Sabat et al. (19). Although preliminary results were promising, several EIEC isolates that had been previously classified by biochemical, serological, and PCR-based methods were found to lack ECA75F. This finding raised concerns regarding the reliability of ECA75F as an *E. coli*-specific marker. These observations indicated that further refinement of the assay design was necessary to improve diagnostic accuracy.

An *in silico* analysis of publicly available genome sequences revealed that approximately 35% of *E. coli* strains lacked ECA75F, whereas it was present in some *Shigella* strains (Table 1). Notably, the ECA75F-carrying *Shigella* strains were identified exclusively from genome sequence data (31) and were found to be genetically distinct from clinical *Shigella* isolates (Fig. 1). Additionally, certain *E. coli* strains lacking ECA75F but positive for *ipaH* were isolated from individuals without dysentery (33). Collectively, these findings suggest that among *ipaH*-positive isolates obtained from symptomatic patients, ECA75F-negative strains are most consistent with *Shigella* spp., whereas ECA75F-positive strains more likely represent EIEC.

Notably, *Shigella* is considered specifically adapted to humans and other primates (7). A recent study (34) suggested that the V1 region of the 16S rRNA gene, containing the ECA75F sequence, may influence translational efficiency and contribute to host adaptation, potentially facilitating the emergence of human-adapted *Shigella* strains. Our findings are consistent with this hypothesis, as the clinical *Shigella* isolates analyzed in this study uniformly lacked the ECA75F sequence. Consequently, the absence of ECA75F in PCR assays could serve as a useful diagnostic marker for screening *Shigella* spp. in clinical specimens.

The phylogenetic analysis identified a small group of ECA75F-positive strains forming the ECA75F-b cluster, which included *Shigella boydii* ST1767 and two environmental isolates annotated as *Shigella* in public databases. This lineage lies outside the major clinical *Shigella* clonal complexes described previously. Analysis of publicly available short-read data confirmed that ST1767 carries the ECA75F sequence within the 16S rRNA gene. The environmental isolates in this cluster exhibited genomic characteristics more closely related to *E. coli*, suggesting atypical features or ambiguous taxonomic placement. These findings highlight the genetic diversity within the *Shigella*/EIEC complex and indicate that some publicly available genomes may include inconsistencies in taxonomic assignment, underscoring the blurred boundary between *Shigella* and EIEC and the importance of integrating molecular assays with lineage-based analyses for accurate interpretation.

In contrast, *ipaH*, which is present in multiple copies on both the virulence plasmid and chromosomes, is a well-established molecular marker of tissue invasiveness (35). In this study, comparative analyses of publicly available genomes identified a conserved region within *ipaH*. Based on this observation, a DpRT-PCR assay was developed in this study using the TaqMan MGB probe system to simultaneously detect ECA75F and *ipaH* (Table 3).

To further elucidate phylogenetic relationships among the isolates, MLST was performed on *Shigella* and *E. coli* isolates using allele sequence data. Although MLST provides lower resolution than whole-genome sequencing (36), it remains a rapid and cost-effective approach for inferring genetic relationships among closely related taxa such as *Shigella* spp. and *E. coli*. Moreover, MLST profiles can be readily derived from WGS data using automated pipelines, allowing the lineage-based framework described here to be directly integrated into WGS-based workflows. Consistent with this framework, MLST analysis in the present study revealed that ECA75F-carrying *Shigella* strains were genetically distinct from clinical *Shigella* isolates (Fig. 1). These findings indicate that combining MLST and DpRT-PCR can enhance classification accuracy, particularly in cases where routine phenotypic diagnostic methods yield ambiguous results.

To evaluate the broader utility of MLST, 42 *Shigella* and 32 *E. coli* isolates (comprising 28 EIEC and 4 non-EIEC *E. coli*, including 8 isolates that were difficult to classify) were analyzed. Based on the resulting STs (Fig. 1), a phylogenetic tree was constructed in which 88 labels, each representing a unique ST–serotype combination (Fig. 2). All *Shigella* isolates initially classified by biochemical and serological testing clustered within the *Shigella* lineage, whereas EIEC isolates formed genetically distinct clusters separate from *Shigella*.

Among the 1,366 *E. coli* strains lacking ECA75F identified in the *in silico* analysis (Table 1), 2 *ipaH*-positive strains belonging to ST270 were located within the EIEC lineage cluster (EIEC-a) (Fig. 2). Although such strains would be classified as *Shigella* by DpRT-PCR, their MLST profiles exclude them from the *Shigella* lineage. Their isolation from individuals without dysenteric symptoms suggests that they may represent atypical EIEC variants, highlighting the additional phylogenetic resolution provided by MLST in distinguishing *Shigella*, EIEC, and non-pathogenic *E. coli*. Similarly, the three reclassified EIEC strains were ECA75F-negative and *ipaH*-positive and clustered within the *Shigella* CC245 lineage by MLST (Fig. 2 and Table 4), consistent with their identification as *Shigella* spp. by DpRT-PCR and demonstrating the assay’s diagnostic value when phenotypic methods yield ambiguous results. In contrast, 4 *E. coli* isolates (2 OUT and 2 O44) were *ipaH*-negative and ECA75F-positive (Table 4) and were therefore classified as *E. coli* by DpRT-PCR. However, their recovery from patients with suspected shigellosis suggests potential etiological involvement. Phylogenetic analysis showed that 3 of these isolates clustered within the ECA75F-a group, which includes ECA75F-positive *Shigella* strains, whereas one belonged to the EIEC-b cluster (Fig. 2). Although the absence of *ipaH* suggests that they are likely nonpathogenic, the possibility of virulence plasmid loss cannot be excluded, as *ipaH*-negative EIEC isolates have been reported in patients with diarrheal symptoms (37). Overall, these findings highlight the diagnostic challenges in phenotype-based differentiation of *Shigella* spp., EIEC, and atypical *E. coli* and underscore the need for reliable molecular tools such as the DpRT-PCR assay.

Given this phylogenetic complexity, we further evaluated the performance of the DpRT-PCR assay using additional clinically derived *Shigella* and EIEC isolates identified by biochemical and serological methods (Table 4). All 174 isolates previously identified as *Shigella* by biochemical and serological testing were consistently identified by DpRT-PCR. Among the 60 EIEC isolates initially classified as EIEC, 3 (EC517, EC579, and EC580; Fig. 2) were reclassified as *Shigella*, whereas 4 were determined to be non-EIEC *E. coli* (Table 4). These results support the high diagnostic specificity of the DpRT-PCR assay, particularly for the identification of *Shigella*, while highlighting minor heterogeneity among EIEC isolates. No cross-reactivity was observed with other enteric species tested, further confirming the specificity of the assay.

Despite its advantages, the DpRT-PCR assay has some limitations. First, the ECA75F sequence, located within the V1 region of the 16S rRNA gene, may influence ribosomal function, though this remains speculative. Second, the taxonomic relationship between *Shigella* and *E. coli* remains debated (12, 13), necessitating cautious interpretation of DpRT-PCR results, particularly without supporting MLST data. Third, previously described EIEC OUT strains, which were ECA75F-negative, are unlikely to be EIEC and may instead represent variant lineages derived from *S. flexneri*, warranting further investigation.

van den Beld et al. (38) reported that no current culture-based or phenotypic diagnostic method can reliably differentiate *Shigella* spp. from EIEC. Based on their exploratory analysis of risk factors associated with shigellosis and EIEC infection, they recommend a combined diagnostic strategy integrating phenotypic and molecular approaches tailored to clinical and epidemiological contexts. Consistent with these recommendations, we propose the DpRT-PCR assay as a valuable diagnostic tool to complement existing biochemical and serological methods for rapid and effective identification of *Shigella* spp. and EIEC in both clinical and public health settings. In addition, the integration of lineage-based approaches such as MLST with molecular diagnostics may facilitate more accurate interpretation of genomic data and improve our understanding of the evolutionary relationships within the *Shigella*/EIEC complex.

## SUPPLEMENTAL MATERIAL

Supplemental materials spectrum.03988-25-s0001.zip FigureS1.pptx, TableS1-S3.xlsx, and TextS1_46STs.fas

ASM does not own the copyrights to Supplemental Material that may be linked to, or accessed through, an article. The authors have granted ASM a non-exclusive, world-wide license to publish the Supplemental Material files. Please contact the corresponding author directly for reuse.
