# Supplementary material for: Genome-based re-evaluation of the ECA75F sequence and development of a dual-probe real-time PCR assay for differentiating Shigella species and enteroinvasive Escherichia coli
Source: Microbiol Spectr. 2026 Jun 15;14(7):e03988-25. doi: 10.1128/spectrum.03988-25 (PMC13340235; doi:10.1128/spectrum.03988-25)
Supplement: Supplemental materials — FigureS1.pptx, TableS1-S3.xlsx, and TextS1_46STs.fas [file spectrum.03988-25-s0001.zip › FigureS1.pptx]

## Slide 1
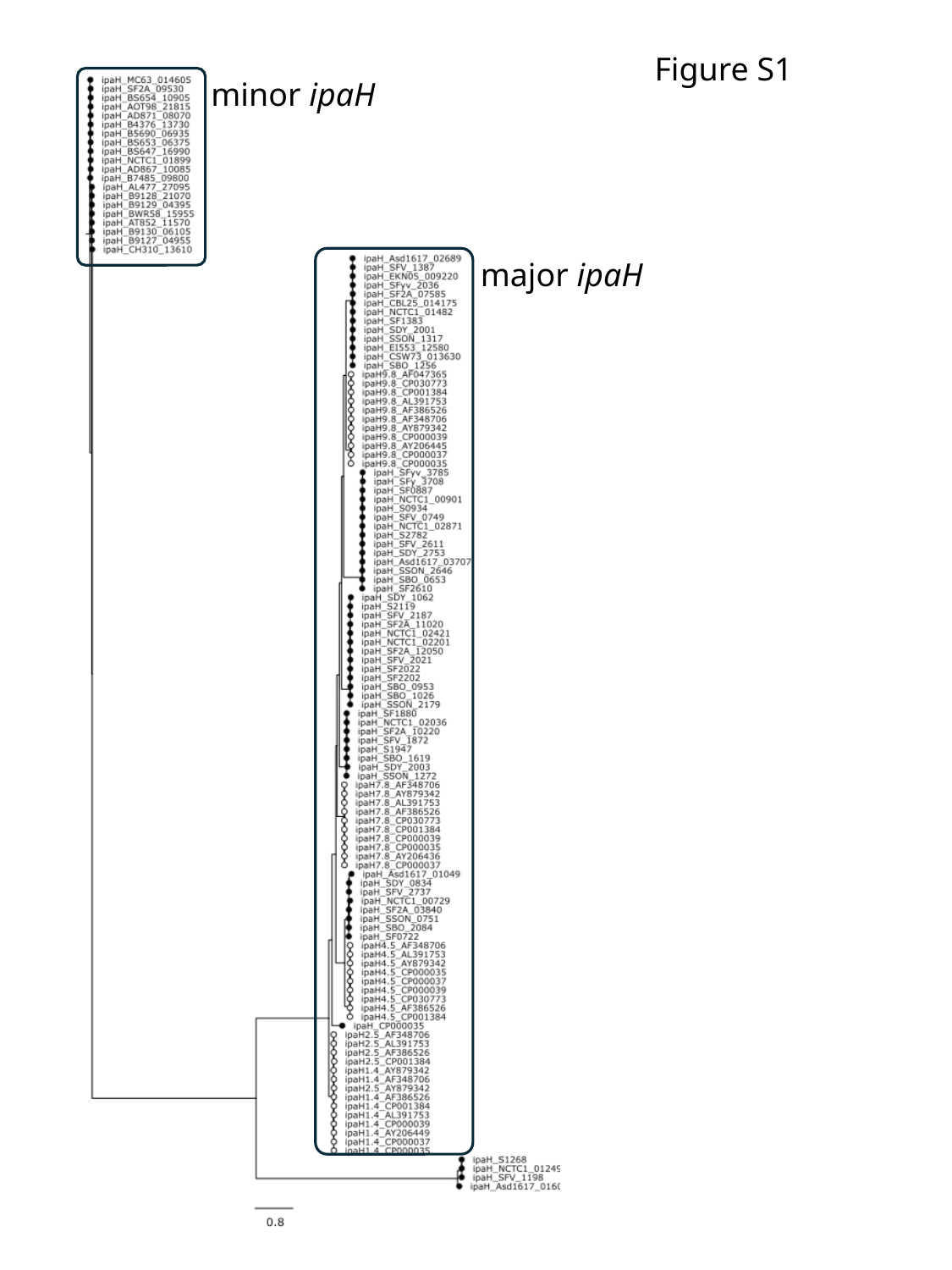

Figure S1
minor ipaH
major ipaH

## Slide 2
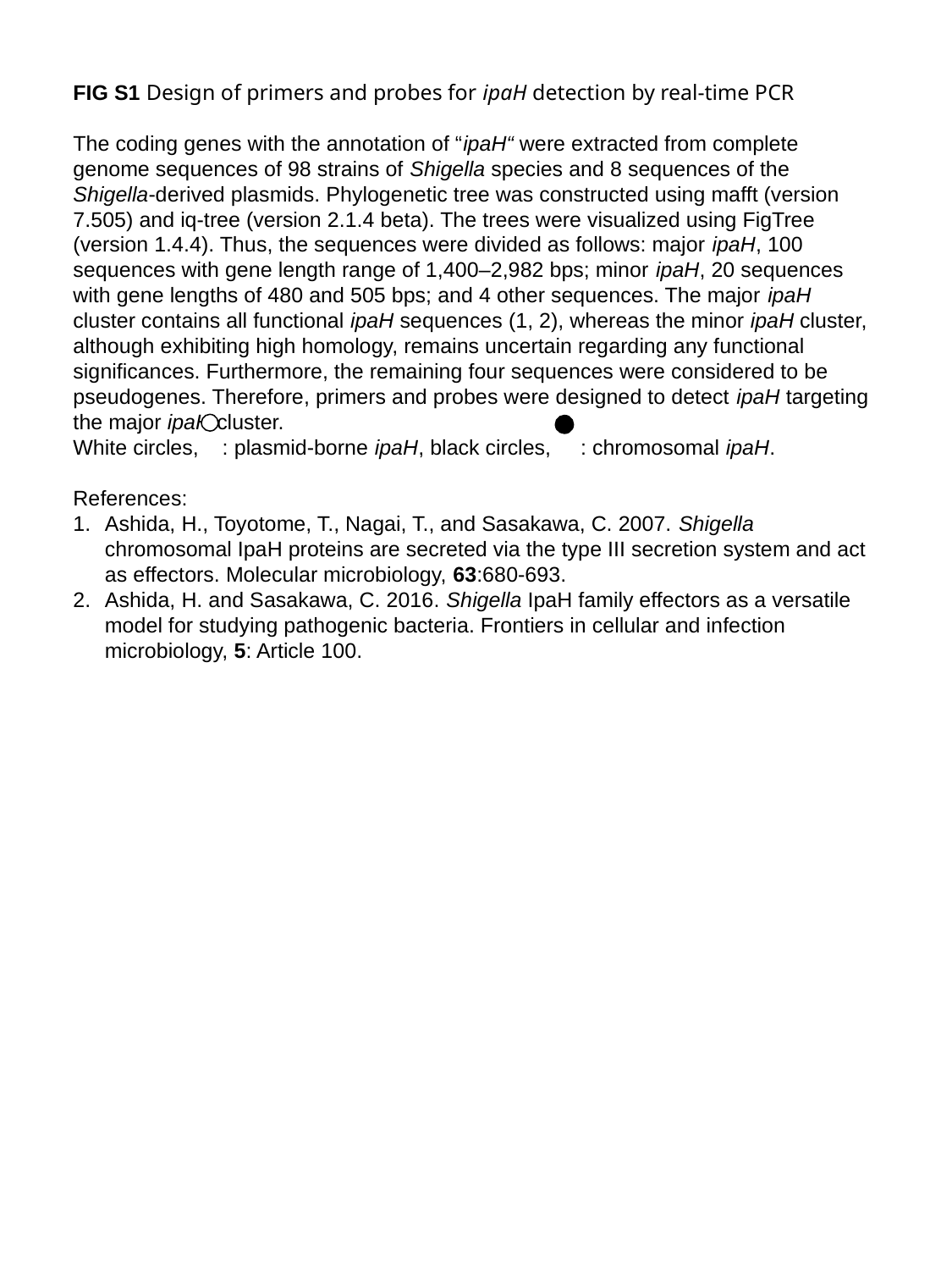

FIG S1 Design of primers and probes for ipaH detection by real-time PCR
The coding genes with the annotation of “ipaH“ were extracted from complete genome sequences of 98 strains of Shigella species and 8 sequences of the Shigella-derived plasmids. Phylogenetic tree was constructed using mafft (version 7.505) and iq-tree (version 2.1.4 beta). The trees were visualized using FigTree (version 1.4.4). Thus, the sequences were divided as follows: major ipaH, 100 sequences with gene length range of 1,400–2,982 bps; minor ipaH, 20 sequences with gene lengths of 480 and 505 bps; and 4 other sequences. The major ipaH cluster contains all functional ipaH sequences (1, 2), whereas the minor ipaH cluster, although exhibiting high homology, remains uncertain regarding any functional significances. Furthermore, the remaining four sequences were considered to be pseudogenes. Therefore, primers and probes were designed to detect ipaH targeting the major ipaH cluster.
White circles, : plasmid-borne ipaH, black circles, : chromosomal ipaH.
References:
Ashida, H., Toyotome, T., Nagai, T., and Sasakawa, C. 2007. Shigella chromosomal IpaH proteins are secreted via the type III secretion system and act as effectors. Molecular microbiology, 63:680-693.
Ashida, H. and Sasakawa, C. 2016. Shigella IpaH family effectors as a versatile model for studying pathogenic bacteria. Frontiers in cellular and infection microbiology, 5: Article 100.
